# Temporal Shifts in Pathogen Profiles Due to the COVID-19 Pandemic in a Romanian Pediatric Tertiary Hospital

**DOI:** 10.3390/children12091258

**Published:** 2025-09-18

**Authors:** Dan Dumitru Vulcanescu, Iulia Cristina Bagiu, Monica Susan, Virgiliu Bogdan Sorop, Octavia Harich, Andrada Oprisoni, Radu Galis, Florin George Horhat

**Affiliations:** 1 Doctoral School, “Victor Babes” University of Medicine and Pharmacy, 300041 Timisoara, Romania; dan.vulcanescu@umft.ro; 2 Department of Microbiology, “Victor Babes” University of Medicine and Pharmacy, 300041 Timisoara, Romania; bagiu.iulia@umft.ro (I.C.B.); horhat.florin@umft.ro (F.G.H.); 3 Multidisciplinary Research Center on Antimicrobial Resistance (MULTI-REZ), Microbiology Department, “Victor Babes” University of Medicine and Pharmacy, 300041 Timisoara, Romania; 4 Clinical Laboratory, “Louis Turcanu” Emergency Hospital for Children, 300011 Timisoara, Romania; 5 Centre for Preventive Medicine, Department of Internal Medicine, Victor Babes University of Medicine and Pharmacy, 300041 Timisoara, Romania; susan.monica@umft.ro; 6 Department of Obstetrics and Gynecology, “Victor Babes” University of Medicine and Pharmacy, 300041 Timisoara, Romania; bogdan.sorop@umft.ro; 7 Department of Functional Sciences, “Victor Babes” University of Medicine and Pharmacy Timisoara, 300041 Timisoara, Romania; harich.octavia@umft.ro; 8 Department of Pediatrics, Discipline of Pediatric Oncology and Hematology, “Victor Babes” University of Medicine and Pharmacy, 300041 Timisoara, Romania; oprisoni.licinia@umft.ro; 9 Department of Neonatology, Clinical County Emergency Hospital Bihor, 410167 Oradea, Romania; 10 Department of Neonatology, Poznan University Medical Sciences, 60-512 Poznan, Poland

**Keywords:** COVID-19, pandemic, pediatric, microbial, trends, Romania

## Abstract

**Highlights:**

**What are the main findings?**

- Pandemic year (2021) profiles were ICU-weighted, with higher proportional detection of non-fermenters and ICU-linked taxa—notably *Pseudomonas aeruginosa*, *Stenotrophomonas maltophilia*, *Enterobacter* spp., and *Candida*—while the rest of the hospital wards showed persistent elevation of *Escherichia coli* and *Klebsiella pneumoniae.*
- The post-pandemic period (2023) showed a community rebound, with renewed detection of group A *Streptococcus* and normalization of outpatient respiratory samples, while several ICU-weighted signals receded toward baseline.

**What is the implication of the main finding?**

- Surveillance should be ward-mix aware: it should prioritize ICU fungal/Gram-negative vigilance during service disruptions, and track community streptococcal resurgence as outpatient activity returns.
- IRR-based, setting-stratified monitoring is a practical way for clinical labs to detect real-time shifts in pathogen mix without duplicating full baseline tables, supporting rapid infection prevention and testing panel adjustments.

**Abstract:**

**Background**: The COVID-19 pandemic disrupted pediatric healthcare systems globally, altering infection dynamics, hospital admissions, and antimicrobial practices. This study aimed to evaluate temporal shifts in patient demographics, clinical aspects, and microbial pathogen profiles in a tertiary pediatric hospital in Western Romania, spanning pre-pandemic (2019), pandemic (2021), and post-pandemic (2023) periods. **Methods**: A retrospective observational study was conducted at the “Louis Țurcanu” Emergency Children’s Hospital, Timișoara. Pediatric patients (<18 years) with laboratory-confirmed bacterial infections were included. Data on demographics, hospital wards, sample types, and pathogen distribution were analyzed using Χ^2^ tests, incidence rate ratios (IRR), and non-parametric statistical methods. **Results**: A total of 3530 patients and 6885 samples were analyzed. Pediatric admissions declined by nearly 50% during the pandemic. The Outpatient and Emergency department observed a decrease in cases, while the ICU and surgical ward cases increased proportionally. Nasal and pharyngeal samples declined during the pandemic, while catheter, blood, and conjunctival samples rose. The study identified a significant shift in pathogen prevalence, with *Escherichia coli* and *Staphylococcus aureus* as the most frequent isolates. ICU patients showed increased rates of *Pseudomonas aeruginosa*, *Candida albicans*, and *Klebsiella pneumoniae*. Group A *Streptococcus* resurged post-pandemic after a decline in 2021. **Conclusions**: The pandemic significantly impacted pediatric infection profiles, hospital service utilization, and sample collection patterns. Strengthening infection surveillance, ensuring consistent reporting standards, and adapting pediatric care to future crises are critical for improving child health outcomes.

## 1. Introduction

Since the emergence of SARS-CoV-2 in late 2019, the COVID-19 pandemic has profoundly disrupted global healthcare systems. Declared a pandemic by the World Health Organization (WHO) in March 2020, COVID-19 prompted extensive public health interventions, including lockdowns, school closures, and widespread reallocation of medical resources [1]. In Romania, the pandemic strained an already under-resourced healthcare system, resulting in fluctuating infection rates, delayed routine care, and substantial challenges in maintaining pediatric services. Early reports, both international and national, suggested that children were largely spared from severe illness, but as the pandemic evolved, pediatric infections increased, highlighting the need to better understand the pandemic’s indirect impact on child health services [2,3].

One area of particular interest is the impact of the pandemic on pediatric bacterial infections. Shifts in healthcare delivery, reduced outpatient visits, and altered diagnostic priorities during the pandemic likely influenced both the detection and management of bacterial infections in children. However, data on how pediatric pathogen prevalence evolved during and after the pandemic, particularly in Eastern Europe, are scarce. Most published studies have focused on adult populations, resistance profiles, or hospital-associated infections, with limited attention to community and ward-level pathogen distribution in pediatric cohorts. Moreover, few studies have compared pre-, during-, and post-pandemic periods in the same population, making it difficult to assess long-term shifts in infection patterns. While Romanian studies have documented the epidemiology of antimicrobial resistance and healthcare-associated infections, the broader trends in bacterial infection dynamics, independent of resistance phenotypes, have not been systematically analyzed [4,5].

A systematic review from the end of 2024 of Romanian healthcare settings indicated notable shifts in bacterial infection patterns during the pandemic period. Gram-negative organisms, particularly *E. coli* and *K. pneumoniae*, emerged as the most frequently isolated pathogens, across 87 studies, encompassing over 20,000 cases. The study also identified *S. aureus* and *Enterococcus* spp. as the leading Gram-positive agents, with a high burden of infections reported in intensive care units (ICUs) and respiratory wards. *Acinetobacter* spp. and *Pseudomonas* spp., contributing significantly to nosocomial infections, and *C. difficile*-associated diarrhea further complicated patient management [6].

This study aims to address the gap in pediatric outcomes related to the COVID-19 pandemic by examining temporal changes in bacterial pathogen profiles in a tertiary pediatric hospital in Western Romania. The main objective is to identify variations in pathogen distribution across three distinct time points, before (2019), during (2021), and after (2023) the COVID-19 pandemic. This analysis is designed as a complementary investigation to a related study focusing on antimicrobial resistance patterns within the same pediatric cohort [7].

## 2. Materials and Methods

### 2.1. Study Design and Data

This retrospective observational study was conducted at the Louis Țurcanu Emergency Children’s Hospital in Timișoara, a tertiary pediatric center serving Western Romania. The analysis included patient records from three distinct time periods: 2019 (pre-pandemic), 2021 (pandemic), and 2023 (post-pandemic). Data were obtained from hospital medical records and institutional databases, ensuring a comprehensive overview of patient demographics, infection profiles, and sample details. The study encompassed all hospital wards, including inpatient units (ICU, Surgery, Pediatrics) and outpatient services, without departmental restrictions. Within ‘Pediatrics,’ four general pediatrics wards are administratively labeled Pediatrics Clinic I–IV, while Other inpatient departments (e.g., Pediatric Surgery/Orthopedics, ENT, Ophthalmology, Plastic Surgery, Nephrology, Pneumology/TB/Infectious Diseases, Neurology, Psychiatry, Dialysis) were analyzed under their recorded department names. For infection trend analyses, we report results across four strata: (1) the total cohort—all settings, (2) Outpatients and the Emergency Department (ED)—non-hospitalized, (3) ICU and NICU—intensive care services, and (4) all other inpatient non-ICU wards—hospitalized.

The inclusion criteria were pediatric patients (under 18 years of age) with complete demographic information and laboratory-confirmed microbial infections. Patients aged over 18 years old, or with incomplete demographic or microbiological data, or those without laboratory-confirmed infections, were excluded from the analysis.

For each eligible patient, the following demographic data were collected: year of admission (within the defined periods), age, sex, location of residence, hospital ward, and length of hospital stay. In this study, the following age categories were used: infant (<2 years), preschool (2–5 years), school (6–12 years), and adolescent (12–18 years). This operational classification reflects our hospital’s reporting standards and was also applied in our previously published article. Demographic characteristics of the pediatric cohort are summarized in our companion paper and are not used as covariates here; they are cited for context only [7]. We distinguish unique patients (de-duplicated across the full study window) from patient-year counts (non-mutually exclusive) and samples (processed clinical specimens). Year-specific patient numbers are reported as patient-year tallies for descriptive context and therefore can exceed the unique total.

Microbiological data included the type of clinical sample and the identified pathogen. Diagnosed infections encompassed a wide range of conditions, including urinary tract infections, respiratory tract infections (upper and lower), wound and skin infections, gastrointestinal and intra-abdominal infections, bloodstream infections, device-associated infections, otitis, ocular infections (conjunctivitis), pleural infections, umbilical infections (omphalitis), central nervous system infections (meningitis), and other less common infection types. Pathogen identification followed hospital protocols, using conventional culture and biochemical methods, or the automated Vitek 2 Compact system. As our objective was to compare setting-specific distributions and laboratory workload, we therefore retained all clinically significant samples without episode-level de-duplication.

This study is part of a broader series examining the impact of the COVID-19 pandemic on pediatric infections in Romania. While previous work from this research project has focused on antimicrobial resistance patterns in the same pediatric cohort, the present study provides a complementary analysis of infection trends, emphasizing changes in pathogen distribution and clinical settings across the pandemic timeline. The percentages of the following data have already been published in the sister study: patient demographics, ward distribution, sample distribution, and pathogen identification rates for the total samples. Similarly, length of stay was already analyzed in the same sister paper [7]. To avoid duplication, this manuscript reports year-to-year changes using incidence rate ratios (IRRs) and includes the underlying period-specific counts in the main tables, reproducing totals previously found in the Supplementary Materials and/or sister article solely as contextual denominators. Together, these studies aim to offer a comprehensive understanding of how the pandemic has reshaped pediatric infectious disease landscapes.

### 2.2. Statistical Analysis

Data were compiled into a spreadsheet and analyzed using MedCalc Statistical Software, version 20.218 (MedCalc Software bv, Ostend, Belgium). A minimum required sample size was calculated using G*Power (version 3.1.9.7), applying an a priori test for a small effect size (0.1) with a power of 95%, yielding a minimum of 1548 patients. Analysis of continuous variables was described in the previous paper [7]. For the purpose of this paper, IRR analysis with 95% confidence intervals (CIs) was performed to compare infection rates across time points: 2019 vs. 2021, 2019 vs. 2023, and 2023 vs. 2021. For IRR interpretation, a value close to 1 indicated no difference in incidence; values >1 indicated a higher rate in the first year, while values <1 indicated a higher rate in the second year.

### 2.3. Ethics

This study was conducted in accordance with the ethical principles outlined in the Declaration of Helsinki. To protect patient confidentiality and comply with ethical standards, no personal identifiers were collected or included in the analysis. As part of doctoral research, the study protocol was reviewed and approved by the Ethics Committee of the “Victor Babeș” University of Medicine and Pharmacy, Timișoara, Romania, which maintains research collaboration with the Louis Țurcanu Emergency Children’s Hospital. Approval was granted under protocol number 31/29.04.2024. Informed consent for laboratory testing was obtained from all patients or their legal guardians at the time of care, in accordance with national guidelines.

## 3. Results

### 3.1. IRR Expansion of Previously Published Ward and Specimen Distributions

**Figure 1:**
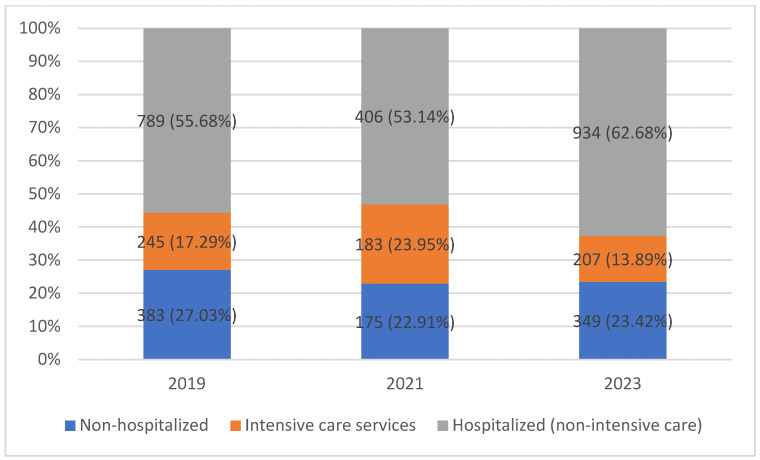
Number of patients and samples by ward category (non-hospitalized, hospitalized non-ICU, ICUs) for 2019, 2021, and 2023.

In total, 6885 samples were processed from 3530 unique patients; the difference reflects routine multi-site and serial sampling in clinical care. Year-specific patient totals sum to 3671 as some patients contributed in more than 1 year (non-mutually exclusive patient-year counts), a difference of 141 patient-year instances. Figure 1 visualizes the workload by setting. Setting distributions shifted across periods. ICU services increased in 2021 (17.29% to 23.95%) with a concurrent decline in non-hospitalized care (27.03% to 22.91%), followed by partial normalization in 2023 for non-hospitalized care (22.91% to 23.43%), while ICU services dropped to 13.89%. Detailed ward-level counts are available in the sister article’s Supplementary Materials Table S1. [7].

Patient numbers dropped sharply during the pandemic (765 cases, 21.67%) compared to the pre-pandemic period (1417 cases, 40.14%), then rebounded post-pandemic (1489 cases, 42.18%). Similarly, sample counts declined during the pandemic (1672, 24.28%) and increased again post-pandemic (2730, 39.65%). Detailed percentage data, including the pre-pandemic baseline of patient demographics, ward and sample distributions, and the LoS analysis are available in the sister study [7]. In the present manuscript, we extend those findings by reporting new IRR analyses to quantify year-to-year changes for the specific wards and samples distribution.

Regarding changes in specific wards, the IRR analysis revealed several significant temporal trends, as shown in Table S1. The statistically significant modifications are further described. Outpatient infections were lower in both 2021 and 2023 compared to 2019 (IRR 2019/2021 = 1.28; IRR 2019/2023 = 1.21). ICU infections peaked during the pandemic (IRR 2019/2021 = 0.68) and fell by 2023 (IRR 2023/2021 = 0.58). Surgery cases peaked in 2021 (IRR 2019/2021 = 0.62) and declined by 2023 (IRR2023/2021 = 0.65), with a similar trend for Dialysis (IRR 2019/2021 = 0.16; IRR 2023/2021 = 0.26). Pediatrics Clinic III peaked in 2023 (IRR 2023/2021 = 3.00), while the pandemic observed a decrease (IRR 2019/2021 = 1.91). Nephrology and Allergology departments also reached their highest infection rates in the post-pandemic period (IRR 2023/2021= 1.99 and 2.65, respectively). Conversely, the Neonatal ICU (NICU) showed the lowest infection rates post-pandemic (IRR 2023/2021 = 0.55), while the Neonatology and Cardiology departments demonstrated a significant difference between 2019 and 2023 (IRR 2019/2023 = 0.57 and 0.42, respectively). Pediatrics Clinic II recorded the lowest rate during the pandemic (IRR 2019/2021 = 2.58), while the ENT department had its highest infection rate in the pre-pandemic period (IRR 2019/2021 = 3.24). Similarly, the ED and Pneumology services had their lowest rates in 2019 (IRR 2019/2021 = 0.33 and 0.20, respectively). Due to limited sample numbers, IRR analysis could not be reliably conducted for Pediatrics Clinic I and the Infectious Diseases department.

Regarding specific samples, the IRR analysis is shown in Table S2. The statistically significant modifications are further described. Nasal secretions declined in 2021 (IRR 2019/2021 = 1.46) and partially rebounded in 2023 (IRR 2023/2021 = 1.23). Wound secretions, hypopharyngeal aspirates, and catheter samples were less frequent pre-pandemic compared to both the pandemic and post-pandemic periods, with IRRs of 0.56, 0.74, and 0.43 respectively for 2019 vs. 2021, while afterwards falling back to pre-pandemic numbers, as evidenced by the IRRs of 0.66, 0.76 and 0.62 respectively for 2023 vs. 2021. Pharyngeal exudates exhibited a distinct pattern, dropping during the pandemic (IRR 2019/2021 = 7.12) and then exceeding pre-pandemic values (IRR 2023/2021 = 10.86). Blood and conjunctival samples were more frequent post-pandemic (IRR 2023/2021 = 1.56, and 1.57, respectively). Pleural fluid samples declined post-pandemic (IRR 223/2021 = 0.49). Cerebrospinal fluid (CSF) samples decreased directly from the pre-pandemic to the post-pandemic period (IRR 2019/2023 = 2.36), while oral lesions increased during the pandemic (IRR 2019/2021 = 0.11) and further increased in 2023, as compared to the pre-pandemic period (IRR 2019/2023 = 0.05). For male genital secretions, a complete comparison across all three time points was not possible due to limited case numbers.

### 3.2. Microbial Identification

Percentages pertaining to identified pathogens from the total samples have been published previously [7], with the most important pathogens being *E. coli*, *S. aureus*, *P. aeruginosa*, *K. pneumoniae*, and group A *Streptococcus*. By applying the grouping algorithm described in the Methods section, grouped analysis can be observed in the Supplementary Materials Tables S3–S5. For hospitalized patients in wards other than ICU or NICU, a similar ranking to total samples was observed, with *E. coli* (21.05%) being the most frequent, followed by *S. aureus* (9.70%), *K. pneumoniae* (9.59%), *S. pneumoniae* (8.39%) and group A *Streptococcus* (8.39%). For patients admitted to intensive care services, however, the ranking was as follows: *P. aeruginosa* (17.53%), followed by *E. coli* (11.40%), *C. albicans* (9.72%), *K. pneumoniae* (9.36%) and *S. maltophilia* (8.64%). The ranking also differed for Outpatient groups, with *S. aureus* (45.17%) followed by group A *Streptococcus* (21.95%), *E. coli* (9.46%), *S. pneumoniae* (9.17%) and *K. pneumoniae* (3.51%). Further, IRR analysis for each category is described and shown in Table 1, Table 2, Table 3 and Table 4.

For the total samples, the following statistically significant trends were observed: a decrease between the pandemic period and the post-pandemic period for *S. aureus*, other *Candida* spp. and other *Serratia* spp.; a maximum during the pandemic, followed by the pre-pandemic period and a minimum during the post-pandemic period for: *P. aeruginosa*; a maximum in 2019, comparable to both 2021 and 2023 for *S. pneumoniae*; a minimum during the pandemic followed by the pre-pandemic period, with a maximum in 2023 for group A *Streptococcus*; a maximum during the pandemic, when compared to the other periods for *C. albicans*, *S. maltophilia*, *Enterobacter* spp.; a maximum in 2023, when compared to both other periods for CoNS and other *Enterococcus* spp.; a minimum in 2023, when compared to the other periods for *C. parapsilosis*, *S. marcescens*, *C. tropicalis* and bacteria registered as Other; an increase between the pandemic and post-pandemic periods for other *Streptococcus* spp.; a minimum in the pre-pandemic period, comparable to the other two periods for *E. faecium*,; a decrease between 2019 and the other timeframes for *P. mirabilis* and *S. paucimobilis*; a downwards trend between 2019 and 2023 for *Salmonella* spp. For *Chryseobacterium* spp. and *H. influenzae*, a proper analysis could not be performed due to low counts.

For samples collected from hospitalized patients in wards other than the ICU or NICU, ratio analysis showed the following statistically significant trends: a maximum value for 2021, when compared to both 2019 and 2023, for *E. coli*, *P. aeruginosa*, *Enterobacter* spp. *S. maltophilia*; a maximum value for 2019 when compared to both other time periods for *S. pneumoniae*; a minimum during the pandemic, followed by the pre-pandemic period and then the post-pandemic period for group A *Streptococcus*; a maximum during the post-pandemic period, when compared to both other timeframes for CoNS and *Enterococcus* spp.; a downward trend between 2019 and 2021 for *P. mirabilis*; a minimum during the post-pandemic period, when compared to both other timeframes for *S. marcescens*, *C. parapsilosis*, *C. tropicalis* and other *Candida* spp.; a minimum in the pre-pandemic period when compared to 2021 and 2023 for *E. faecium*. For *Chryseobacterium* spp. *S. paucimobilis* and *H. influenzae*, a proper analysis could not be performed due to low counts.

For samples collected from non-hospitalized patients, the following statistically significant observations were made: a maximum value for the pandemic period, as compared to both 2019 and 2023 for *S. aureus*; a minimum during the pandemic, followed by the pre-pandemic and then by the post-pandemic period for group A *Streptococcus*, a minimum during 2023, as compared to the other datapoints for *E. coli*; a maximum during the pre-pandemic period for *K. pneumoniae*; an upwards trend between 2019 and 2021 for *S. pneumoniae*; a downward trend between 2019 and 2021 for *P. mirabilis*. The following could not be properly assessed due to low counts: *C. parapsilosis*, *Morganella* spp., other *Streptococcus* spp., other *Acinetobacter* spp., *Enterobacter* spp., *E. faecalis*, other *Klebsiella* spp., other *Candida* spp., *E. faecium*, other *Enterococcus* spp., *H. influenzae*, other *Pseudomonas* spp., *S. marcescens*.

For samples collected from patients admitted to intensive care services, the following statistically significant observations were made: a maximum value for the pandemic for *C. albicans*; an upward trend between 2021 and 2023 for CoNS; a minimum for 2019, when compared to both 2021 and 2023 for *S. maltophilia*; a downward trend between 2021 and 2023 for *C. parapsilosis*; a downward trend between 2019 and 2023 for *E. faecium*; a maximum in 2019, as compared to 2021 for other *Klebsiella* spp.; a maximum for 2019, when compared to both 2021 and 2023 for *S. paucimobilis*; a maximum in 2023, when compared to both pre-pandemic and pandemic periods for other *Enterococcus* spp. Data could not be properly compared due to low counts for *Chryseobacterium* spp., other *Proteus* spp., other *Serratia* spp., *H. influenzae*, *Morganella* spp., *Salmonella* spp., group A *Streptococcus* and other *Streptococcus* spp.

## 4. Discussion

### 4.1. Overview

The COVID-19 pandemic disrupted pediatric care in Romania through delays, reduced access, and a shift toward more empirical antibiotic use; together with prolonged hospitalizations, increased invasive procedures, and broader-spectrum therapies in children, these factors likely fostered multidrug-resistant (MDR) organisms [8,9,10,11]. Our sister study detailed sex, origin, and age patterns [7], identifying infants as the most affected, which is consistent with findings reported by Rossato et al. [12], and documented hospitalization declines that mirror the findings of Wilde et al. [13]. Admissions fell by roughly 50% (from 1417 in 2019 to 765 in 2021), with recovery in 2023 (1489), against a backdrop of stable or increased microbiological sampling. This combination suggests both closer inpatient monitoring during peak restrictions and renewed community pathogen circulation as measures eased.

During the 2021 pandemic peak, pediatric admissions declined sharply, with outpatient visits 21.87% lower than in 2019. The ICU-weighted case mix in 2021 and the rebound of non-hospitalized activity in 2023 (Figure 1) explain much of the observed specimen-profile shift and the organism signals we quantify by incidence rate ratios (IRR), indicating a service-mix effect rather than wholesale pathogen replacement. Specifically, ICU, Surgery, and Dialysis admissions increased: ICU rates were ~1.5× higher in 2021 than in 2019 or 2023 (0.68 and 0.58, respectively), Surgery was ~1.6× higher (0.62 and 0.65), and Dialysis showed notable shifts (0.16 in 2019 vs. 2021; 0.26 in 2023 vs. 2021). Nationally, similar declines were seen in pediatric admissions, with ICU cases comprising a larger share during pandemic peaks [6,14,15,16,17]. Ward-specific fluctuations shaped microbiological sampling patterns because specimen types map closely to clinical services (e.g., blood cultures in ICU, urine in nephrology, respiratory samples in outpatients), paralleling national and international findings [6,18,19,20,21].

In our laboratory stream, the ICU-weighted 2021 profile coincided with proportionally more blood and lower-respiratory samples and fewer outpatient upper-respiratory submissions; by 2023, as services normalized, we observed a rebound of pharyngeal/nasal specimens alongside higher blood and conjunctival sampling (Table S2). Because specific specimen types align with specific departments (e.g., blood in ICU, urine in nephrology, respiratory in outpatients), these ward fluctuations naturally reshaped the mix of samples received.

The organism profile tracked these service-mix shifts. In 2021, signals for ICU-associated taxa, such as non-fermenting Gram-negative rods, selected Enterobacterale and *Candida* spp. were relatively more prominent, consistent with more invasive sampling and greater critical-care exposure. In 2023, with outpatient activity restored, community-linked pathogens re-emerged, most notably group A *Streptococcus,* and *Streptococcus pneumoniae* rose from its 2021 trough, in line with renewed respiratory submissions. These patterns are captured by our IRR estimates in Table 1, Table 2, Table 3 and Table 4 and are best interpreted in light of the changing service mix rather than as evidence of wholesale pathogen replacement. National trends reported elsewhere similarly show fewer pediatric admissions with proportionally greater ICU burdens during pandemic peaks [6,14,15,16,17,18,19,20,21]

### 4.2. Bacterial Trends

The microbial analysis of pediatric infections revealed distinct shifts in the prevalence of bacterial pathogens over the study period. Gram-negative bacilli, particularly *E. coli*, *K. pneumoniae* and *P. aeruginosa*, remained among the most frequently isolated pathogens in hospitalized children. These results are consistent with previous studies, including work by Goff et al., who reported that pandemic-related disruptions to antimicrobial stewardship programs led to increased nosocomial transmission of multidrug-resistant Gram-negative bacteria [6,22]. Prior reports describe well-established seasonal patterns and characteristic pathogen profiles in pediatric infections, such as winter peaks of otitis media, streptococcal pharyngitis, and pneumococcal disease, stable burdens of urinary tract infections dominated by *E. coli* and *K. pneumoniae*; and frequent *S. pneumoniae*/*S. aureus* in respiratory/skin infections, while opportunistic pathogens such as *P. aeruginosa* and *Candida* spp. are largely concentrated in hospital settings and among critically ill children [23,24,25,26,27,28,29,30].

Studies from Europe, Asia, and the Americas reported marked declines in pediatric infections, with hospital admissions for infectious diseases in Romania dropping, as well. Notably, respiratory bacterial infections with *S. pneumoniae* or *H. influenzae* saw a steep decline due to reduced viral co-infections and limited social contact [6,16], while community-acquired infections such as otitis media, scarlet fever, and acute tonsillitis greatly diminished in 2020 [14,31]; nosocomial infections, however, among hospitalized children became more severe [6]. Fungal infections, particularly *Candida* bloodstream infections, remained a concern in ICUs, with some centers reporting spikes due to prolonged hospital stays and broad-spectrum antibiotic use [32]. In our dataset, we observe the same direction of change: 2021 shows contraction of outpatient activity with a relative shift toward ICU submissions and blood/lower-respiratory specimens, followed in 2023 by a recovery of outpatient respiratory sampling and renewed detection of community-linked pathogens. Additionally, 2021 was the peak for both *C. albicans* (8.32% in 2019 → 13.27% in 2021 → 7.40% in 2023) and *C. parapsilosis* (4.83% → 6.02% → 2.80%), with the IRR calculations being significant for *C. albicans* (2019 vs. 2021; 2023 vs. 2021) and for *C. parapsilosis* (2023 vs. 2021).

However, among hospital isolates, fungal infections were less common (under 10%), while *H. influenzae*, *E. coli*, and *K. pneumoniae* are common Gram-negatives (over 50%) [33,34,35]. Frequent Gram-positive bacteria include *S. pneumoniae* and *S. aureus* and *Enterococcus* spp. However, Romania’s pre-pandemic burden of resistant hospital pathogens was high; it had Europe’s highest incidence of MRSA bloodstream infections in 2019 and one of the highest for carbapenem-resistant *Klebsiella*, underscoring that *S. aureus* and *Klebsiella*/*E. coli* were prevalent threats, even before COVID-19 [23,32]. Additionally, *S. aureus* displayed a pandemic-era peak, particularly in outpatient settings, before experiencing a post-pandemic decline. This suggests that the increased use of antibiotics and infection control measures during the pandemic may have temporarily elevated the detection rates of *S. aureus* in non-hospitalized populations before a subsequent decrease due to improved management practices [6,23].

As COVID-19 restrictions eased, pediatric infection rates rebounded. Group A *Streptococcus* infections experienced an unexpected resurgence in late 2022, with some countries noting a sevenfold increase in invasive cases among young children [16,31]. Pneumococcal infections, which had plummeted in 2020, rebounded significantly in 2022 and 2023, with some regions reporting higher-than-baseline incidence. Outpatient pediatric infections surged in 2023, with clinics reporting increased cases of pharyngitis, otitis media, and skin infections [31,36]. Hospital infections returned to pre-pandemic levels, although ICU settings continued to see a higher proportion of MDR bacterial infections, particularly *K. pneumoniae* and *P. aeruginosa* [6,37,38].

External factors likely shaped these trends: in 2021, widespread COVID-19 non-pharmaceutical interventions, such as lockdowns, masking, school closures, hand hygiene, and distancing curtailed transmission, aligning with international reports of sharp drops in pediatric RSV, influenza, and streptococcal pharyngitis [39,40]. Our data similarly show fewer outpatient samples and lower levels of *S. pyogenes* that year. However, prior studies note that these measures may have limited typical early-life microbial exposures (“immunity debt”), helping to explain post-restriction rebound, including increased levels of invasive *S. pyogenes* and *H. influenzae* in several European countries [34,41,42,43]. Consistent with this, we observed recovery of outpatient pharyngeal sampling in 2023, with renewed detection of community-linked pathogens (e.g., group A *Streptococcus*). Lockdown-related changes in activity, social contact, and diet have also been associated with reduced gut microbial diversity and altered immune responses in children [44,45,46].

Although AMR was not the focus here and was addressed in the companion study [7], it likely shaped observed trends, given our country’s high MDR burden and pandemic era antibiotic overuse; healthcare-associated pathogens (*K. pneumoniae*, *P. aeruginosa*) were prominent, and post-pandemic data underscore ongoing prevention/control needs [28,31,47]. In pediatrics, ~70% of isolates were resistant to ≥1 class, with pandemic peaks for cephalosporins (42.91%), combination agents (40.95%), and reserve antibiotics (38.89%); MRSA, MRCoNS, and CRO surged in 2021, while ESBL declined thereafter. Resistant infection was also associated with pediatric abandonment, showing higher resistance to fluoroquinolones, aminoglycosides, and reserve agents among abandoned children [7]. Additionally, neonates of mothers with vaginal dysbiosis/infections, often admitted to NICU/preterm wards, are especially vulnerable to *Candida* spp. and Gram-negatives like *K. pneumoniae* and *Pseudomonas* spp. [48,49]. These findings support integrated antimicrobial stewardship and child protection strategies in post-pandemic planning.

### 4.3. Limitations and Perspectives

This study has several limitations, inherent to its retrospective, single-center design. While the dataset provides valuable insights into pediatric infection trends over a five-year period, its findings may not fully generalize to the broader Romanian pediatric population or to other healthcare systems. The study reflects the specific patient mix, diagnostic practices, and infection control policies of a single tertiary pediatric hospital, which may differ from national or regional patterns. Also, as the same underlying dataset was partly presented in our previously published descriptive study, this analysis offers complementary findings by extending prior descriptive work through IRR-based temporal comparisons.

Additionally, while comprehensive, the dataset excludes cases with incomplete demographic or microbiological information, potentially introducing selection bias. Infection with SARS-CoV-2 was not systematically recorded or analyzed, and its potential role as a confounding factor in co- or superinfections during the pandemic period must be acknowledged.

Regarding the methodology, the IRR estimates should be interpreted as descriptive comparisons of setting- and period-specific distributions rather than as causal effects, as no formal sensitivity testing was performed. This restricts explanatory insight and should be considered when interpreting our findings

Another important limitation is the reduction in the number of microbiological samples collected during the pandemic year (2021), which may introduce selection bias. Due to restricted outpatient activity and prioritization of severe inpatient cases, mild or community-acquired infections may have been underrepresented. This could have led to an overestimation of nosocomial or resistant pathogens and reduced the statistical robustness of trend analyses, particularly for rarer organisms. The lower sample count may also affect the generalizability of 2021-specific findings, necessitating cautious interpretation when comparing across years.

Pathogen prevalence was reported in aggregate rather than stratified by sample type, anatomical site, or specific clinical syndromes. While this approach allows for an overview of trends across years, it limits conclusions about infection sources or clinical relevance in specific contexts (e.g., bloodstream vs. respiratory infections). Also, several patients produced multiple samples; however, the totals reflect laboratory workload rather than the number of distinct infection episodes, consistent with our objective to compare distributions across care settings.

Similarly, the absence of detailed clinical data—such as comorbidities, severity scores, and outcomes—restricts interpretation of infection trends in relation to patient risk factors and clinical outcomes. Additionally, the lack of ICU sub-classification can also be viewed as a potential limitation. Available hospital data only permitted general categorization into ‘ICU’ and ‘neonatal ICU’, without distinction between pediatric medical, surgical, or other ICU types. This may have limited the granularity of our infection setting comparisons.

Finally, the study highlights the broader challenge of inconsistent reporting standards in microbiological surveillance across Romania. This variability complicates inter-study comparisons and underscores the need for standardized national reporting systems to better inform infection control policies and antimicrobial stewardship programs.

Future multicentric studies with standardized methodologies and inclusion of molecular and clinical outcome data are essential for a more comprehensive understanding of pediatric infection trends and resistance dynamics in Romania.

## 5. Conclusions

This study provides a structured assessment of the impact of the COVID-19 pandemic on pediatric microbial infections across 2019, 2021, and 2023 in a Romanian tertiary hospital. Our findings highlight significant changes in pathogen prevalence, with hospitalization trends emphasizing the pandemic’s profound influence on both medical and social aspects of child healthcare. Pathogen incidence varied across settings and years, with *E. coli* and *S. aureus* maintaining stable prevalence, while *Streptococcus* group A showed a marked resurgence post-pandemic. Certain pathogens, including *C. albicans*, *Enterobacter* spp., and *S. maltophilia*, peaked during the pandemic across hospital wards and ICU/NICU settings. Non-ICU wards experienced gradual pathogen shifts, whereas outpatient samples indicated distinct infection patterns, with *S. aureus* increasing and *Streptococcus* group A rebounding post-pandemic.

## Figures and Tables

**Table 1:** Pathogen ratio analysis for total pathogens (hospitalized, non-hospitalized and intensive care services).

Species	*n* (2019/2021/2023)	2019 vs. 2021	2019 vs. 2023	2023 vs. 2021
Total	2483/1669/2733			
Gram-positive				
*S. aureus*	360/255/346	0.95 (0.80–1.13; 0.5641)	1.14 (0.98–1.34; 0.0937)	0.83 (0.70–0.99; 0.0358) *
CoNS	119/70/190	1.14 (0.85–1.55; 0.3798)	0.69 (0.54–0.87; 0.0019) *	1.66 (1.26–2.20; 0.0004) *
*S. pneumoniae*	199/72/186	1.86 (1.41–2.45; <0.0001) *	1.18 (0.96–1.45; 0.1244)	1.58 (1.20–2.09; 0.0013) *
*Streptococcus* group A	191/14/381	9.19 (5.32–15.86; <0.0001) *	0.55 (0.46–0.66; <0.0001) *	16.67 (9.74–28.50; <0.0001) *
*Streptococcus* group B	5/5/11	0.67 (0.19–2.33; 0.5323)	0.50 (0.17–1.44; 0.1990)	1.35 (0.47–3.88; 0.5810)
*Streptococcus* spp. (other)	12/3/17	2.69 (0.76–9.56; 0.1252)	0.78 (0.37–1.63; 0.5026)	3.47 (1.02–11.80; 0.0472) *
*E. faecalis*	58/26/53	1.50 (0.94–2.40; 0.0875)	1.20 (0.83–1.75; 0.3354)	1.25 (0.78–2.00; 0.3580)
*E. faecium*	29/39/65	0.50 (0.31–0.81; 0.0051) *	0.49 (0.32–0.76; 0.0015) *	1.02 (0.68–1.53; 0.9201)
*Enterococcus* spp. (other)	15/17/83	0.59 (0.30–1.19; 0.1432)	0.20 (0.11–0.35; <0.0001) *	2.99 (1.77–5.06; <0.0001) *
Gram-negative				
*E. coli*	415/313/442	0.89 (0.76–1.05; 0.1630)	1.03 (0.89–1.19; 0.6664)	0.86 (0.74–1.01; 0.0700)
*K. pneumoniae*	229/138/227	1.12 (0.90–1.39; 0.3229)	1.11 (0.92–1.34; 0.2888)	1.01 (0.81–1.26; 0.9474)
*Klebsiella* spp. (other)	28/12/18	1.57 (0.80–3.10; 0.1922)	1.71 (0.94–3.10; 0.0769)	0.92 (0.44–1.91; 0.8206)
*P. mirabilis*	85/26/65	2.20 (1.41–3.43; 0.0005) *	1.44 (1.04–1.99; 0.0298) *	1.53 (0.97–2.42; 0.0688)
*Proteus* spp. (other)	5/3/2	1.12 (0.27–4.70; 0.8746)	2.75 (0.53–14.10; 0.2271)	0.41 (0.07–2.45; 0.3268)
*Enterobacter* spp.	43/46/40	0.63 (0.41–0.96; 0.0309) *	1.18 (0.77–1.82; 0.4503)	0.53 (0.35–0.82; 0.0039) *
*Citrobacter* spp.	14/7/13	1.35 (0.54–3.34; 0.5211)	1.18 (0.56–2.52; 0.6618)	1.14 (0.45–2.86; 0.7840)
*Salmonella* spp.	4/4/15	0.67 (0.17–2.70; 0.5764)	0.29 (0.10–0.88; 0.0294) *	2.30 (0.76–6.93; 0.1401)
*S. marcescens*	57/40/22	0.96 (0.64–1.44; 0.8432)	2.85 (1.74–4.67; <0.0001) *	0.34 (0.20–0.57; <0.0001) *
*Serratia* spp. (other)	2/6/1	0.22 (0.05–1.11; 0.0675)	2.20 (0.20–4.27; 0.5201)	0.10 (0.01–0.85; 0.0347) *
*Morganella* spp.	9/3/2	2.02 (0.55–7.47; 0.2921)	1.41 (0.53–3.80; 0.4928)	1.43 (0.37–5.53; 0.6053)
*P. aeruginosa*	224/187/197	0.81 (0.66–0.99; 0.0388) *	1.25 (1.02–1.53; 0.0279) *	0.65 (0.50–0.80; <0.0001) *
*Pseudomonas* spp. (other)	5/5/15	0.67 (0.19–2.33; 0.5323)	0.37 (0.13–1.01; 0.0523)	1.84 (0.67–5.06; 0.2396)
*A. baumannii*	34/22/39	1.04 (0.61–1.79; 0.8849)	0.96 (0.60–1.52; 0.8577)	1.09 (0.64–1.84; 0.7593)
*Acinetobacter* spp. (other)	10/8/15	0.84 (0.33–2.14; 0.7170)	0.73 (0.33–1.63; 0.4478)	1.15 (0.49–2.71; 0.7526)
*S. maltophilia*	44/85/58	0.35 (0.24–0.50; <0.0001) *	0.83 (0.56–1.24; 0.3687)	0.42 (0.30–0.59; <0.0001) *
*S. paucimobilis*	18/2/2	6.06 (1.40–6.15; 0.0157) *	9.90 (2.29–42.60; 0.0021) *	0.61 (0.09–4.35; 0.6241)
*Chryseobacterium* spp.	9/14/-	0.43 (0.19–1.00; 0.0507)	NA	NA
Fungi				
*C. albicans*	122/129/135	0.64 (0.49–0.82; 0.0005) *	0.99 (0.77–1.28; 0.9600)	0.64 (0.50–0.82; 0.0005) *
*C. parapsilosis*	65/53/28	0.83 (0.57–1.19; 0.3081)	2.55 (1.63–3.99; <0.0001) *	0.32 (0.20–0.51; <0.0001) *
*C. tropicalis*	17/14/8	0.82 (0.40–1.66; 0.5785)	2.34 (1.01–5.42; 0.0483)	0.35 (0.15–0.84; 0.0181) *
*Candida* spp. (other)	36/37/27	0.66 (0.41–1.04; 0.0735)	1.47 (0.89–2.42; 0.1353)	0.45 (0.27–0.74; 0.0016) *
Other	20/17/9	0.79 (0.41–1.52; 0.4821)	2.44 (1.11–5.38; 0.0264)	0.32 (0.14–0.73; 0.0064) *

Values are presented as IRR (95% CI; *p*-value), with CIs rounded to 2 decimal places and *p*-values to 4 decimal places; *: statistically significant, NA: not applicable. Period-specific isolate counts are reproduced here from the companion article’s Supplementary Materials Table S1, solely as contextual denominators [7].

**Table 2:** Pathogen ratio analysis for pathogens of hospitalized patients.

Species	*n* (2019/2021/2023)	2019 vs. 2021	2019 vs. 2023	2023 vs. 2021
Total	1438/914/1842			
Gram-positive				
*S. aureus*	139/92/176	0.96 (0.73–1.27; 0.7798)	1.01 (0.8–1.28; 0.9259)	0.95 (0.73–1.24; 0.7084)
CoNS	77/46/150	1.07 (0.73–1.55; 0.7413)	0.66 (0.50–0.87; 0.0037) *	1.62 (1.15–2.28; 0.0053) *
*S. pneumoniae*	165/53/134	1.98 (1.44–2.73; <0.0001) *	1.58 (1.24–2.00; 0.0002) *	1.26 (0.91–1.74; 0.1720)
*Streptococcus* group A	104/10/238	6.62 (3.44–12.73; <0.0001) *	0.56 (0.44–0.71; <0.0001) *	11.83 (6.25–22.38; <0.0001) *
*Streptococcus* group B	1/1/7	0.64 (0.04–10.19; 0.7493)	0.18 (0.02–1.49; 0.1122)	3.48 (0.43–28.32; 0.2439)
*Streptococcus* spp. (other)	10/2/15	3.18 (0.70–14.55; 0.1357)	0.85 (0.38–1.91; 0.6991)	3.73 (0.85–16.34; 0.0809)
*E. faecium*	12/20/35	0.38 (0.19–0.78; 0.0088) *	0.44 (0.23–0.85; 0.0144) *	0.87 (0.5–1.52; 0.6223)
*E. faecalis*	50/19/47	1.67 (0.98–2.86; 0.0588)	1.36 (0.91–2.04; 0.1341)	1.23 (0.72–2.11; 0.4523)
*Enterococcus* spp. (other)	10/13/60	0.49 (0.21–1.12; 0.0910)	0.21 (0.11–0.42; <0.0001) *	2.29 (1.25–4.20; 0.0071) *
Gram-negative				
*E. coli*	287/231/365	0.79 (0.65–0.96; 0.0165) *	1.01 (0.85–1.19; 0.9387)	0.79 (0.65–0.94; 0.0096) *
*K. pneumoniae*	139/91/172	0.97 (0.74–1.28; 0.8403)	1.03 (0.82–1.31; 0.7755)	0.94 (0.72–1.23; 0.6453)
*Klebsiella* spp. (other)	13/9/14	0.92 (0.39–2.16; 0.8465)	1.19 (0.56–2.54; 0.6548)	0.77 (0.33–1.79; 0.5488)
*P. mirabilis*	57/19/56	1.91 (1.13–3.23; 0.0159) *	1.30 (0.90–1.90; 0.1666)	1.46 (0.87–2.48; 0.1552)
*Proteus* spp. (other)	1/3/2	0.21 (0.02–2.04; 0.1796)	0.64 (0.06–7.07; 0.7158)	0.33 (0.06–1.99; 0.2267)
*Enterobacter* spp.	28/36/27	0.49 (0.30–0.82; 0.0059) *	1.33 (0.78–2.26; 0.2974)	0.37 (0.22–0.62; 0.0001) *
*Citrobacter* spp.	10/5/12	1.27 (0.43–3.74; 0.6608)	1.07 (0.46–2.48; 0.8802)	1.19 (0.42–3.40; 0.7412)
*Salmonella* spp.	4/3/12	0.85 (0.19–3.80; 0.8298)	0.43 (0.14–1.33; 0.1410)	1.99 (0.56–7.06; 0.2880)
*S. marcescens*	43/24/15	1.14 (0.69–1.89; 0.6119)	3.67 (2.03–6.63; <0.0001) *	0.31 (0.16–0.60; 0.0004) *
*Serratia* spp. (other)	1/3/1	0.21 (0.02–2.04; 0.1796)	1.28 (0.08–20.49; 0.8614)	0.17 (0.02–1.59; 0.1197)
*Morganella* spp.	5/1/7	3.18 (0.37–27.28; 0.2911)	0.91 (0.29–2.89; 0.8788)	3.48 (0.43–28.32; 0.2439)
*P. aeruginosa*	104/91/107	0.73 (0.54–0.98; 0.0333) *	1.24 (0.94–1.64; 0.1240)	0.58 (0.44–0.78; 0.0003) *
*Pseudomonas* spp. (other)	2/3/10	0.42 (0.07–2.54; 0.3480)	0.26 (0.06–1.17; 0.0789)	1.66 (0.45–6.03; 0.4440)
*A. baumannii*	14/9/26	0.99 (0.43–2.3; 0.9809)	0.69 (0.36–1.32; 0.2645)	1.44 (0.67–3.08; 0.3522)
*Acinetobacter* spp. (other)	4/3/6	0.85 (0.19–3.8; 0.8298)	0.85 (0.24–3.03; 0.8064)	0.99 (0.25–3.98; 0.9932)
*S. maltophilia*	13/23/7	0.36 (0.18–0.71; 0.0034) *	2.38 (0.95–5.97; 0.0654)	0.15 (0.06–0.35; <0.0001) *
*S. paucimobilis*	4/1/-	2.54 (0.25–125.35; 0.4417)	NA	NA
*Chryseobacterium* spp.	4/1/-	2.54 (0.25–125.35; 0.4417)	NA	NA
*H. influenzae*	-/-/9	NA	NA	NA
Fungi				
*C. albicans*	64/52/94	0.78 (0.54–1.14; 0.2016)	0.87 (0.63–1.21; 0.4075)	0.90 (0.63–1.27; 0.5463)
*C. parapsilosis*	31/19/14	1.04 (0.58–1.85; 0.8987)	2.83 (1.50–5.35; 0.0013) *	0.37 (0.18–0.73; 0.0046) *
*C. tropicalis*	22/16/14	0.72 (0.28–1.86; 0.4931)	3.84 (1.04–14.21; 0.0438) *	0.19 (0.05–0.70; 0.0132) *
*Candida* spp. (other)	11/8/6	0.87 (0.46–1.67; 0.6867)	2.01 (1.03–3.95; 0.0420) *	0.43 (0.21–0.89; 0.0237) *
Other	64/52/94	0.87 (0.35–2.18; 0.7746)	2.35 (0.87–6.36; 0.0935)	0.37 (0.13–1.08; 0.0684)

Values are presented as IRR (95% CI; *p*-value), with CIs rounded to 2 decimal places and *p*-values to 4 decimal places; *: statistically significant, NA: not applicable. Period-specific isolate counts and percentages are detailed in Supplementary Materials Table S3.

**Table 3:** Pathogen ratio analysis for pathogens of non-hospitalized patients (ED and Outpatients).

Species	*n* (2019/2021/2023)	2019 vs. 2021	2019 vs. 2023	2023 vs. 2021
Total	444/192/389			
Gram-positive				
*S. aureus*	195/123/145	0.69 (0.52–0.91; 0.0087) *	1.18 (0.91–1.52; 0.2064)	0.58 (0.43–0.78; 0.0003) *
CoNS	7/3/6	1.01 (0.26–3.94; 0.9897)	1.02 (0.34–3.07; 0.9688)	0.99 (0.24–3.99; 0.9855)
*S. pneumoniae*	30/17/47	0.76 (0.41–1.42; 0.3917)	0.56 (0.35–0.90; 0.0171) *	1.36 (0.76–2.44; 0.2944)
*Streptococcus* group A	87/4/134	9.41 (3.40–25.99; <0.0001) *	0.57 (0.42–0.77; 0.0003) *	16.53 (6.03–45.37; <0.0001) *
*Streptococcus* group B	3/2/2	0.65 (0.11–3.91; 0.6369)	1.31 (0.22–7.91; 0.7654)	0.49 (0.07–3.53; 0.4818)
*Streptococcus* spp. (other)	2/1/-	0.87 (0.05–51.03; 0.8771)	NA	NA
*E. faecalis*	1/2/-	NA	NA	NA
*E. faecium*	-/-/1	NA	NA	NA
*Enterococcus* spp. (other)	-/-/4			
Gram-negative		NA	NA	NA
*E. coli*	49/23/25	0.92 (0.55–1.56; 0.7588)	1.72 (1.04–2.83; 0.0343) *	0.54 (0.30–0.97; 0.0393) *
*K. pneumoniae*	31/2/3	6.70 (1.59–28.29; 0.0096) *	9.05 (2.75–29.85; 0.0003) *	0.74 (0.12–4.47; 0.7431)
*Klebsiella* spp. (other)	1/-/-			
*P. mirabilis*	15/2/4	3.24 (0.73–14.32; 0.1205)	3.29 (1.08–9.98; 0.0359) *	0.99 (0.18–5.44; 0.9881)
*Enterobacter* spp.	1/-/1	NA	0.88 (0.01–68.77; 0.9340)	NA
*S. marcescens*	-/1/-	NA	NA	NA
*Morganella* spp.	4/1/-	1.73 (0.17–85.19; 0.6902)	NA	NA
*P. aeruginosa*	3/5/6	0.26 (0.06–1.10; 0.0666)	0.44 (0.11–1.76; 0.2454)	0.59 (0.18–1.97; 0.3920)
*Pseudomonas* spp. (other)	-/-/1	NA	NA	NA
*A. baumannii*	1/1/2	0.43 (0.03–6.95; 0.5541)	0.44 (0.04–4.85; 0.5010)	0.99 (0.09–10.95; 0.9916)
*Acinetobacter* spp. (other)	1/-/-	NA	NA	NA
*H. influenzae*	-/-/3	NA	NA	NA
Fungi				
*C. albicans*	8/2/4	1.73 (0.36–8.22; 0.4908)	1.75 (0.52–5.86; 0.3628)	0.99 (0.18–5.44; 0.9881)
*C. parapsilosis*	5/-/-	NA	NA	NA
*Candida* spp. (other)	-/3/1	NA	NA	0.16 (0.02–1.59; 0.1191)

Values are presented as IRR (95% CI; *p*-value), with CIs rounded to 2 decimal places and *p*-values to 4 decimal places; *: statistically significant, NA: not applicable. Period-specific isolate counts and percentages are detailed in Supplementary Materials Table S4.

**Table 4:** Pathogen ratio analysis for patients in the ICU and NICU.

Species	*n* (2019/2021/2023)	2019 vs. 2021	2019 vs. 2023	2023 vs. 2021
Total	601/563/502			
Gram-positive				
*S. aureus*	26/40/25	0.61 (0.37–1.01; 0.0569)	0.87 (0.49–1.52; 0.6134)	0.71 (0.42–1.18; 0.1848)
CoNS	35/21/34	1.57 (0.90–2.72; 0.1115)	0.86 (0.53–1.39; 0.5324)	1.83 (1.05–3.19; 0.0336) *
*S. pneumoniae*	4/2/5	1.88 (0.34–10.31; 0.4670)	0.67 (0.18–2.49; 0.5455)	2.83 (0.55–14.63; 0.2157)
*Streptococcus* group A	-/-/9	NA	NA	NA
*Streptococcus* group B	1/2/2	0.47 (0.04–5.2; 0.5381)	0.42 (0.04–4.60; 0.4744)	1.13 (0.16–8.05; 0.9029)
*Streptococcus* spp. (other)	-/-/2	NA	NA	NA
*E. faecium*	17/19/29	0.84 (0.43–1.63; 0.6098)	0.49 (0.26–0.90; 0.0211) *	1.72 (0.96–3.11; 0.0706)
*E. faecalis*	7/5/6	1.32 (0.42–4.17; 0.6406)	0.97 (0.32–2.91; 0.9575)	1.36 (0.41–4.47; 0.6168)
*Enterococcus* spp. (other)	5/4/19	1.18 (0.31–4.40; 0.8106)	0.22 (0.08–0.59; 0.0027) *	5.37 (1.81–15.88; 0.0024) *
Gram-negative				
*E. coli*	79/59/52	1.26 (0.88–1.80; 0.2055)	1.26 (0.87–1.83; 0.2142)	1.00 (0.67–1.47; 0.9837)
*K. pneumoniae*	59/45/52	1.23 (0.82–1.85; 0.3110)	0.94 (0.64–1.4; 0.7726)	1.31 (0.86–1.98; 0.2096)
*Klebsiella* spp. (other)	14/3/4	4.39 (1.25–15.35; 0.0206) *	2.91 (0.95–8.90; 0.0609)	1.51 (0.34–6.76; 0.5927)
*P. mirabilis*	13/5/5	2.44 (0.87–6.90; 0.0914)	2.16 (0.77–6.11; 0.1452)	1.13 (0.33–3.93; 0.8475)
*Proteus* spp. (other)	4/-/-	NA	NA	NA
*Enterobacter* spp.	14/10/12	1.32 (0.58–2.99; 0.5112)	0.97 (0.44–2.12; 0.9402)	1.36 (0.58–3.17; 0.4814)
*Citrobacter* spp.	4/2/1	1.88 (0.34–10.31; 0.4670)	3.33 (0.37–29.87; 0.2829)	0.57 (0.05–6.25; 0.6415)
*Salmonella* spp.	-/1/3	NA	NA	3.39 (0.35–32.70; 0.2911)
*S. marcescens*	14/15/7	0.88 (0.42–1.83; 0.7282)	1.66 (0.67–4.15; 0.2754)	0.53 (0.21–1.3; 0.1659)
*Serratia* spp. (other)	1/3/-	0.31 (0.03–3.02; 0.3156)	NA	NA
*Morganella* spp.	-/1/-	NA	NA	NA
*P. aeruginosa*	117/91/84	1.21 (0.90–1.63; 0.2111)	1.16 (0.85–1.57; 0.3426)	1.04 (0.76–1.44; 0.7962)
*Pseudomonas* spp. (other)	3/2/4	1.41 (0.23–8.47; 0.7071)	0.62 (0.14–2.80; 0.5381)	2.26 (0.41–12.39; 0.3477)
*A. baumannii*	19/12/11	1.49 (0.72–3.09; 0.2867)	1.44 (0.68–3.05; 0.3447)	1.04 (0.45–2.37; 0.9335)
*Acinetobacter* spp. (other)	5/5/9	0.94 (0.27–3.26; 0.9225)	0.46 (0.15–1.39; 0.1689)	2.03 (0.68–6.11; 0.2058)
*S. maltophilia*	31/62/51	0.47 (0.30–0.73; 0.0009) *	0.51 (0.32–0.80; 0.0038) *	0.93 (0.63–1.37; 0.7131)
*S. paucimobilis*	14/1/2	13.16 (1.73–100.42; 0.0129) *	5.82 (1.32–25.75; 0.0202) *	2.26 (0.2–25.0; 0.5061)
*Chryseobacterium* spp.	5/13/-	0.36 (0.13–1.02; 0.0547)	NA	NA
*H. influenzae*	-/-/1	NA	NA	NA
Fungi				
*C. albicans*	50/75/37	0.63 (0.43–0.91; 0.0148) *	1.12 (0.72–1.75; 0.6029)	0.56 (0.37–0.84; 0.0054) *
*C. parapsilosis*	29/34/14	0.80 (0.48–1.33; 0.3947)	1.72 (0.90–3.30; 0.1001)	0.47 (0.25–0.88; 0.0180) *
*C. tropicalis*	8/6/5	1.25 (0.43–3.64; 0.6775)	1.33 (0.43–4.09; 0.6178)	0.94 (0.29–3.10; 0.9213)
*Candida* spp. (other)	14/18/12	0.73 (0.36–1.48; 0.3860)	0.97 (0.44–2.12; 0.9402)	0.75 (0.36–1.58; 0.4533)
Other	9/9/3	0.94 (0.37–2.39; 0.8965)	2.50 (0.67–9.27; 0.1718)	0.38 (0.10–1.40; 0.1447)

Values are presented as IRR (95% CI; *p*-value), with CIs rounded to 2 decimal places and *p*-values to 4 decimal places; *: statistically significant, NA: not applicable. Period-specific isolate counts and percentages are detailed in Supplementary Materials Table S5.

## Data Availability

Data are available upon request from the corresponding author.

## Supplementary Materials

The following supporting information can be downloaded at: https://www.mdpi.com/article/10.3390/children12091258/s1, Table S1. IRR calculation for specific wards based on total patient number; Table S2. IRR calculation for samples based on total sample number; Table S3. Percentages for pathogens identified in samples from hospitalized patients; Table S4. Percentages for pathogens identified in samples from non-hospitalized patients; Table S5. Percentages for pathogens identified in samples from intensive care services patients.
