# Supplementary material for: Temporal Shifts in Pathogen Profiles Due to the COVID-19 Pandemic in a Romanian Pediatric Tertiary Hospital
Source: Children (Basel). 2025 Sep 18;12(9):1258. doi: 10.3390/children12091258 (PMC12468864; doi:10.3390/children12091258)
Supplement: Supplementary file 1 [file children-12-01258-s001.zip › children-3846816-supplementary.pdf]

children

MDPI

Supplementary material

Table S1. IRR calculation for specific wards- based on total patient number

IRR (95% CI; p)	*n* (2019/ 2021/ 2023)	2019 vs. 2021	2019 vs. 2023	2023 vs. 2021
**Total**	**2483/ 1669/ 2733**			
Outpatient	372/ 157/ 324	1.28 (1.04-1.57; 0.0193) *	1.21 (1.02-1.42; 0.0264) *	1.06 (0.86-1.31; 0.5842)
ICU	179/ 142/ 161	0.68 (0.54-0.86; 0.0015) *	1.17 (0.93-1.46; 0.1754)	0.58 (0.46-0.74; <0.0001) *
Pediatrics IV	151/ 72/ 142	1.13 (0.84-1.52; 0.4080)	1.12 (0.88-1.42; 0.3654)	1.01 (0.75-1.36; 0.9306)
Surgery	136/ 119/ 151	0.62 (0.48-0.80; 0.0003) *	0.95 (0.74-1.21; 0.6568)	0.65 (0.50-0.84; 0.0010) *
Pediatrics III	85/ 24/ 140	1.91 (1.21-3.03; 0.0059) *	0.64 (0.48-0.84; 0.0016) *	3.00 (1.93-4.66; <0.0001) *
Pediatrics I	73/ 40/ -	0.99 (0.66-1.46; 0.9414)	NA	NA
Nephrology	67/ 32/ 124	1.13 (0.74-1.74; 0.5767)	0.57 (0.42-0.77; 0.0003) *	1.99 (1.34-2.96; 0.0007) *
NICU	66/ 42/ 45	0.85 (0.57-1.26; 0.4166)	1.54 (1.05-2.27; 0.0280) *	0.55 (0.36-0.85; 0.0064) *
Preterm	65/ 33/ 51	1.06 (0.69-1.63; 0.7784)	1.34 (0.92-1.95; 0.1256)	0.79 (0.51-1.24; 0.3112)
Oncology	58/ 40/ 65	0.78 (0.52-1.18; 0.2444)	0.94 (0.65-1.35; 0.7270)	0.83 (0.56-1.25; 0.3806)
Neonatology	49/ 39/ 91	0.68 (0.44-1.04; 0.0766)	0.57 (0.40-0.81; 0.0017) *	1.20 (0.82-1.76; 0.3561)
Pediatrics II	43/ 9/ 40	2.58 (1.25-5.32; 0.0103) *	1.13 (0.73-1.75; 0.5843)	2.28 (1.10-4.73; 0.0263) *
Gastroenterology	32/ 24/ 39	0.72 (0.42-1.23; 0.2298)	0.86 (0.54-1.38; 0.5391)	0.83 (0.50-1.40); 0.4929)
Other	31/ 17/ 43	0.98 (0.54-1.79; 0.9591)	0.76 (0.47-1.21; 0.2444)	1.30 (0.74-2.29; 0.3662)
ENT	30/ 5/ 13	3.24 (1.25-8.38; 0.0154) *	2.42 (1.26-4.67; 0.0080) *	1.34 (0.47-3.76; 0.5835)
Hematology	29/ 9/ 22	1.74 (0.82-3.69; 0.1496)	1.39 (0.79-2.42; 0.2532)	1.26 (0.58-2.74; 0.5672)
Nutritional recovery	23/ 7/ 13	1.77 (0.76-4.15; 0.1866)	1.86 (0.94-3.68; 0.0756)	0.95 (0.30-2.40; 0.9206)
Palliative care	22/ 19/ 26	0.63 (0.34-1.16; 0.1376)	0.89 (0.50-1.58; 0.6875)	0.70 (0.39-1.28; 0.2481)
Orthopedics	14/ 4/ 12	1.89 (0.62-5.76; 0.2632)	1.23 (0.57-2.66; 0.6062)	1.54 (0.50-4.80); 0.4550)
Allergology	13/ 6/ 31	1.17 (0.44-3.09; 0.7518)	0.44 (0.23-0.85; 0.0137) *	2.65 (1.10-6.39; 0.0294) *
AIDS	13/ 4/ 14	1.75 (0.57-5.40; 0.3269)	0.98 (0.46-2.08; 0.9494)	1.80 (0.59-5.48; 0.3022)
ED	11/ 18/ 25	0.33 (0.16-0.70;	0.46 (0.23-0.94;	0.71 (0.39-1.32;

		0.0040) *	0.0339) *	0.2799)
Cardiology	8/ 9/ 20	0.48 (0.18-1.25; 0.1324)	0.42 (0.18-0.96; 0.0390) *	1.14 (0.52-2.52; 0.7428)
Pneumology	4/ 11/ 23	0.20 (0.06-0.62; 0.0054) *	0.18 (0.06-0.53; 0.0017) *	1.07 (0.52-2.22; 0.8462)
Dialysis	3/ 10/ 5	0.16 (0.04-0.59; 0.0058) *	0.63 (0.15-2.64; 0.5282)	0.26 (0.09-0.75; 0.0134) *
Infectious diseases	-/ -/ 50	NA	NA	NA

IRR = Incidence Rate Ratio; CI = Confidence Interval; *p* = *p*-value.Values are presented as IRR (95% CI; *p*-value), with CIs rounded to 2 decimal places and *p*-values to 4 decimal places; *: statistically significant, NA: not applicable. Period-specific isolate counts are reproduced here from the companion article's Supplement S1, Table S1.3, solely as contextual denominators [7].

Table S2. IRR calculation for samples- base on total sample number

IRR (95% CI; p)	*n* (2019/ 2021/ 2023)	2019 vs. 2021	2019 vs. 2023	2023 vs. 2021
**Total**	**2483/ 1669/ 2733**			
Urine	718/ 511/ 757	0.95 (0.83-1.08; 0.4014)	1.04 (0.93-1.17; 0.4773)	0.91 (0.80-1.03; 0.1353)
Nasal secretion	377/ 174/ 350	1.46 (1.21-1.76; 0.0001) *	1.18 (1.01-1.38; 0.0328) *	1.23 (1.02-1.49; 0.0330) *
Wound secretion	220/ 265/ 286	0.56 (0.46-0.68; <0.0001) *	0.85 (0.70-1.02; 0.0743)	0.66 (0.55-0.79; <0.0001) *
Hypopharyngeal aspirate	214/ 196/ 244	0.74 (0.60-0.90; 0.0030) *	0.96 (0.80-1.17; 0.7097)	0.76 (0.63-0.93; 0.0071) *
Pharyngeal exudate	201/ 19/ 337	7.12 (9.43-11.45; <0.0001) *	0.66 (0.55-0.79; <0.0001) *	10.86 (6.82-17.31; <0.0001) *
Other	173/ 21/ 28	5.55 (3.51-8.76; <0.0001) *	6.79 (9.54-10.16; <0.0001) *	0.82 (0.46-1.44; 0.4853)
Peritoneal fluid	101/ 90/ 75	0.76 (0.56-1.01; 0.0590)	1.48 (1.09-2.01; 0.0113) *	0.51 (0.37-0.70; <0.0001) *
Blood	96/ 77/ 196	0.84 (0.62-1.14; 0.2629)	0.54 (0.42-0.69; <0.0001) *	1.56 (1.19-2.04; 0.0013) *
Catheter	84/ 133/ 134	0.43 (0.32-0.56; <0.0001) *	0.69 (0.52-0.91; 0.0087) *	0.62 (0.48-0.79; 0.0001) *
Otic secretion	76/ 46/ 69	1.11 (0.77-1.61; 0.5735)	1.21 (0.87-1.69; 0.2561)	0.92 (0.63-1.34; 0.6601)
Conjunctival secretion	48/ 34/ 87	0.95 (0.61-1.48; 0.8231)	0.61 (0.42-0.87; 0.0060) *	1.57 (1.05-2.34; 0.0281) *
Pleural fluid	40/ 34/ 27	0.79 (0.50-1.26; 0.3224)	1.63 (1.00-2.66; 0.0516)	0.49 (0.29-0.81; 0.0055) *
Umbilical secretion	32/ 11/ 34	1.96 (0.98-3.90; 0.0554)	1.03 (0.64-1.68; 0.8902)	1.89 (0.96-3.75; 0.0669)
CSF	30/ 11/ 14	1.84 (0.92-3.67; 0.0859)	2.36 (1.25-4.45; 0.0083) *	0.78 (0.35-1.72; 0.5376)
Puss	21/ 12/ 14	1.18 (0.58-2.40; 0.6513)	1.65 (0.84-3.25; 0.1484)	0.71 (0.33-1.55; 0.3943)
Stool	21/ 13/ 17	1.09 (0.54-2.18; 0.8123)	1.36 (0.71-2.58; 0.3498)	0.80 (0.39-1.65; 0.5482)
Skin	18/ 11/ 15	1.10 (0.52-2.34; 0.8005)	1.32 (0.66-2.62; 0.4294)	0.84 (0.38-1.82; 0.6510)
Vaginal secretion	12/ 4/ 11	2.02 (0.65-6.27; 0.2239)	1.20 (0.53-2.72; 0.6638)	1.68 (0.54-5.30; 0.3726)

Oral lesion	1/ 6/ 20	0.11 (0.01-0.93; 0.0430) *	0.05 (0.01-0.41; 0.0047) *	2.04 (0.82-5.09; 0.1261)
Male genital secretion	-/ 4/ 15	NA	NA	2.30 (0.76-6.93; 0.1401)

IRR = Incidence Rate Ratio; CI = Confidence Interval; *p* = *p*-value. Values are presented as IRR (95% CI; *p*-value), with CIs rounded to 2 decimal places and *p*-values to 4 decimal places; *: statistically significant, NA: not applicable. Period-specific isolate counts are reproduced here from the companion article's Supplement S1, Table S1.4, solely as contextual denominators [7].

Table S3. Percentages for pathogens identified in samples from hospitalized patients.

**Species counts for hospitalized patients**	**2019**	**2021**	**2023**	**Total= 4194**
**Gram positive**				
S. aureus	139 (9.67%)	92 (10.05%)	176 (9.56%)	407 (9.70%)
CoNS	77 (5.35%)	46 (5.03%)	150 (8.15%)	273 (6.51%)
S. pneumoniae	165 (11.47%)	53 (5.79%)	134 (7.28%)	352 (8.39%)
Streptococcus group A	104 (7.23%)	10 (1.09%)	238 (12.93%)	352 (8.39%)
Streptococcus group B	1 (0.07%)	1 (0.11%)	7 (0.38%)	9 (0.21%)
Streptococcus spp. (other)	10 (0.70%)	2 (0.22%)	15 (0.81%)	27 (0.64%)
E. faecium	12 (0.83%)	20 (2.19%)	35 (1.90%)	67 (1.60%)
E. faecalis	50 (3.48%)	19 (2.08%)	47 (2.55%)	116 (2.77%)
Enterococcus spp. (other)	10 (0.70%)	13 (1.42%)	60 (3.26%)	83 (1.98%)
**Gram negative**				
E. coli	287 (19.96%)	231 (25.25%)	365 (19.83%)	883 (21.05%)
K. pneumoniae	139 (9.67%)	91 (9.95%)	172 (9.34%)	402 (9.59%)
Klebsiella spp. (other)	13 (0.90%)	9 (0.98%)	14 (0.76%)	36 (0.86%)
P. mirabilis	57 (3.96%)	19 (2.08%)	56 (3.04%)	132 (3.15%)
Proteus spp. (other)	1 (0.07%)	3 (0.33%)	2 (0.11%)	6 (0.14%)
Enterobacter spp.	28 (1.95%)	36 (3.93%)	27 (1.47%)	91 (2.17%)
Citrobacter spp.	10 (0.70%)	5 (0.55%)	12 (0.65%)	27 (0.64%)
Salmonella spp.	4 (0.28%)	3 (0.33%)	12 (0.65%)	19 (0.45%)
S. marcescens	43 (2.99%)	24 (2.62%)	15 (0.81%)	82 (1.96%)
Serratia spp. (other)	1 (0.07%)	3 (0.33%)	1 (0.05%)	5 (0.12%)
Morganella spp.	5 (0.35%)	1 (0.11%)	7 (0.38%)	13 (0.31%)
P. aeruginosa	104 (7.23%)	91 (9.95%)	107 (5.81%)	302 (7.20%)
Pseudomonas spp. (other)	2 (0.14%)	3 (0.33%)	10 (0.54%)	15 (0.36%)
A. baumannii	14 (0.97%)	9 (0.98%)	26 (1.41%)	49 (1.17%)
Acinetobacter spp. (other)	4 (0.28%)	3 (0.33%)	6 (0.33%)	13 (0.31%)
S. maltophilia	13 (0.90%)	23 (2.51%)	7 (0.38%)	43 (1.03%)
S. paucimobilis	4 (0.28%)	1 (0.11%)		5 (0.12%)
Chryseobacterium spp.	4 (0.28%)	1 (0.11%)		5 (0.12%)
H. influenzae			9 (0.49%)	9 (0.21%)
**Fungi**				
C. albicans	64 (4.45%)	52 (5.68%)	94 (5.11%)	210 (5.01%)
C. parapsilosis	31 (2.16%)	19 (2.08%)	14 (0.76%)	64 (1.53%)
C. tropicalis	9 (0.63%)	8 (0.87%)	3 (0.16%)	20 (0.48%)
Candida spp. (other)	22 (1.53%)	16 (1.75%)	14 (0.76%)	52 (1.24%)
**Other**	11 (0.76%)	8 (0.87%)	6 (0.33%)	25 (0.60%)

Table S4. Percentages for pathogens identified in samples from non-hospitalized patients.

**Species counts for Outpatients and ED**	**2019**	**2021**	**2023**	**Total= 1025**
**Gram positive**				

S. aureus	195 (43.92%)	123 (64.06%)	145 (37.28%)	463 (45.17%)
CoNS	7 (1.58%)	3 (1.56%)	6 (1.54%)	16 (1.56%)
S. pneumoniae	30 (6.76%)	17 (8.85%)	47 (12.08%)	94 (9.17%)
Streptococcus group A	87 (19.59%)	4 (2.08%)	134 (34.45%)	225 (21.95%)
Streptococcus group B	3 (0.68%)	2 (1.04%)	2 (0.51%)	7 (0.68%)
Streptococcus spp. (other)	2 (0.45%)	1 (0.52%)		3 (0.29%)
E. faecalis	1 (0.23%)	2 (1.04%)		3 (0.29%)
E. faecium			1 (0.26%)	1 (0.10%)
Enterococcus spp. (other)			4 (1.03%)	4 (0.39%)
**Gram negative**				
E. coli	49 (11.04%)	23 (11.98%)	25 (6.43%)	97 (9.46%)
K. pneumoniae	31 (6.98%)	2 (1.04%)	3 (0.77%)	36 (3.51%)
Klebsiella spp. (other)	1 (0.23%)			1 (0.10%)
P. mirabilis	15 (3.38%)	2 (1.04%)	4 (1.03%)	21 (2.05%)
Enterobacter spp.	1 (0.23%)		1 (0.26%)	2 (0.20%)
S. marcescens		1 (0.52%)		1 (0.10%)
Morganella spp.	4 (0.90%)	1 (0.52%)		5 (0.49%)
P. aeruginosa	3 (0.68%)	5 (2.60%)	6 (1.54%)	14 (1.37%)
Pseudomonas spp. (other)			1 (0.26%)	1 (0.10%)
A. baumannii	1 (0.23%)	1 (0.52%)	2 (0.51%)	4 (0.39%)
Acinetobacter spp. (other)	1 (0.23%)			1 (0.10%)
H. influenzae			3 (0.77%)	3 (0.29%)
**Fungi**				
C. albicans	8 (1.80%)	2 (1.04%)	4 (1.03%)	14 (1.37%)
C. parapsilosis	5 (1.13%)			5 (0.49%)
Candida spp. (other)		3 (1.56%)	1 (0.26%)	4 (0.39%)

Table S5. Percentages for pathogens identified in samples from intensive care services patients.

Species counts for ICUs	2019	2021	2023	Total= 1666
**Gram positive**				
S. aureus	26 (4.33%)	40 (7.08%)	25 (5.00%)	91 (5.46%)
CoNS	35 (5.82%)	21 (3.72%)	34 (6.80%)	90 (5.40%)
S. pneumoniae	4 (0.67%)	2 (0.35%)	5 (1.00%)	11 (0.24%)
Streptococcus group A			9 (1.80%)	9 (0.12%)
Streptococcus group B	1 (0.17%)	2 (0.35%)	2 (0.40%)	5 (0.30%)
Streptococcus spp. (other)			2 (0.40%)	2 (0.06%)
E. faecium	17 (2.83%)	19 (3.36%)	29 (5.80%)	65 (3.90%)
E. faecalis	7 (1.16%)	5 (0.88%)	6 (1.20%)	18 (1.08%)
Enterococcus spp. (other)	5 (0.83%)	4 (0.71%)	19 (3.80%)	28 (1.68%)
**Gram negative**				
E. coli	79 (13.14%)	59 (10.44%)	52 (10.40%)	190 (11.40%)
K. pneumoniae	59 (9.82%)	45 (7.96%)	52 (10.40%)	156 (9.36%)
Klebsiella spp. (other)	14 (2.33%)	3 (0.53%)	4 (0.80%)	21 (2.16%)
P. mirabilis	13 (2.16%)	5 (0.88%)	5 (1.00%)	23 (1.38%)
Proteus spp. (other)	4 (0.67%)			4 (0.42%)
Enterobacter spp.	14 (2.33%)	10 (1.77%)	12 (2.40%)	36 (1.02%)
Citrobacter spp.	4 (0.67%)	2 (0.35%)	1 (0.20%)	7 (0.66%)
Salmonella spp.		1 (0.18%)	3 (0.60%)	4 (0.24%)
S. marcescens	14 (2.33%)	15 (2.65%)	7 (1.40%)	36 (2.16%)
Serratia spp. (other)	1 (0.17%)	3 (0.53%)		4 (0.24%)
Morganella spp.		1 (0.18%)		1 (0.06%)
P. aeruginosa	117 (19.47%)	91 (16.11%)	84 (16.80%)	292 (17.53%)

Pseudomonas spp. (other)	3 (0.50%)	2 (0.35%)	4 (0.80%)	9 (0.54%)
A. baumannii	19 (3.16%)	12 (2.12%)	11 (2.20%)	42 (2.52%)
Acinetobacter spp. (other)	5 (0.83%)	5 (0.88%)	9 (1.80%)	19 (1.14%)
S. maltophilia	31 (5.16%)	62 (10.97%)	51 (10.20%)	144 (8.64%)
S. paucimobilis	14 (2.33%)	1 (0.18%)	2 (0.40%)	17 (1.26%)
Chryseobacterium spp.	5 (0.83%)	13 (2.30%)		18 (1.08%)
H. influenzae			1 (0.20%)	1 (0.54%)
**Fungi**				
C. albicans	50 (8.32%)	75 (13.27%)	37 (7.40%)	162 (9.72%)
C. parapsilosis	29 (4.83%)	34 (6.02%)	14 (2.80%)	77 (4.62%)
C. tropicalis	8 (1.33%)	6 (1.06%)	5 (1.00%)	19 (1.14%)
Candida spp. (other)	14 (2.33%)	18 (3.19%)	12 (2.40%)	44 (2.64%)
**Other**	9 (1.50%)	9 (1.59%)	3 (0.60%)	21 (1.26%)
